# Empowering Frontline Primary Healthcare Workers in a Global Health Partnership Training of Trainers Intervention to Strengthen the Prevention and Control of Cardiovascular Disease in Mozambique

**DOI:** 10.5334/gh.1052

**Published:** 2022-08-02

**Authors:** Philippa Harris, Edna Juga, Neusa Bay, Chamila Adams, Patrícia Nhatitima, Adjine Mastala, Nilza Matavel, Arminda Mufanequisso, Nelta Mabote, Eunice Mondlane, Naisa Manafe, Paula Pinto, Ros Kirkland, David Mazza, Ana Mocumbi

**Affiliations:** 1 Primary Care International, Oxford, United Kingdom, GB; 2 Instituto Nacional de Saúde, Marracuene, MZ; 3 Mozambique Institute for Health Education and Research, Maputo, MZ; 4 Hospital Geral de Mavalene, Maputo, MZ; 5 Direcção de Saúde da Cidade de Maputo, Maputo, MZ; 6 Universidade Eduardo Mondlane, Faculty of Medicine, MZ

**Keywords:** Global Health Partnership, training of trainers, hypertension, Interprofessional training, Primary Health Care, Non-communicable diseases

## Abstract

**Background:**

Unpreparedness of health professionals to address non-communicable diseases (NCD) at peripheral health facilities is a critical health system challenge in Mozambique. To address this weakness and decentralize NCD care, training of the primary care workforce is needed. We describe our experience in the design and implementation of a cascade training of trainers (ToT) intervention to strengthen the prevention and control of cardiovascular disease.

**Methods:**

Between October 2018 and March 2020 a multidisciplinary global technical partnership was used to train frontline primary care health professionals from a resource-poor suburban setting in Maputo, Mozambique. Following engagement with local policy makers, clinicians, and academics, core training materials were developed, and a ToT cascade was implemented, supported by an on-site pilot clinic. Knowledge and confidence acquisition by participants and new local trainers were assessed using pre- and post-training surveys, while trainees and trainers completed further evaluation surveys at the end of the program.

**Results:**

Three ToT workshops trained 60 mixed cadre healthcare workers in assessment, diagnosis and management of hypertension, diabetes, and cardiovascular risk; of these, 11 became new local trainers. Mean pre- and post-test scores improved in all three training workshops (53% to 90%, 59% to 78%, and 58% to 74% respectively). New local trainers were highly rated by their trainees and reported increased confidence as trainers (mean Likert scale 3.0/5 pre-training to 4.8/5 post-training).

**Conclusion:**

This global health partnership delivered interprofessional training with good knowledge acquisition and increased self-reported confidence. Intensive local supervision and hands-on training empowered a new cohort of trainers to strengthen the prevention and control of cardiovascular disease and is likely to improve coordination and integration at primary care level as well as support the national scale up of NCD care delivery.

## Background

In sub-Saharan Africa (SSA), non-communicable diseases (NCDs) are responsible for one-third of the disability-adjusted life years, with the burden of cardiovascular diseases becoming a critical health issue [1,2]. The majority of Mozambique’s over 28 million inhabitants lives in extreme poverty [3,4], and is witnessing the emergence of new patterns of risk behaviors and health challenges (mainly in urban and peri-urban communities) that relate to an increase in metabolic disease and hypertension (HTN) [5]. The country’s World Health Organization (WHO) supported “STEPwise Approach to Surveillance” (STEPS) survey [6] in 2005 revealed the significant burden of HTN [7], and 10 years later an increasing prevalence in adults aged 25–64 years, from 33.1 to 38.9% (P = 0.048) [8]. However, the low rates of awareness, treatment among the aware, and control among those treated remained largely the same [8].

The public health sector in Mozambique is the main provider of health services on a national scale and is organized into four levels of care (primary – health centres; secondary – district hospitals and general urban hospitals; tertiary – provincial hospitals; quaternary – specialized and sub-specialty hospitals). The country faces one of the most severe workforce shortages in SSA [9], with only a small proportion of specialists [10], contributing to high levels of undiagnosed and untreated HTN. Additionally, guideline application is low, services are ill-equipped, and there is poor integration of risk factor screening and effective management [11]. Both, unpreparedness of health professionals and lack of clinical protocols hamper the system’s readiness to address HTN and DM [12].

Despite strong political commitment and efforts of task-shifting, the lack of opportunities for training is expressed in Mozambique’s NCD National Strategic Plan [13], which involves the implementation of NCD clinics at first referral hospitals, investment in integrated NCD care provision platforms, design of simplified primary care guidelines, and training of frontline health workers to manage these chronic conditions [14].

Training of Trainers (ToT) cascade interventions are a well-known method of increasing the capacity of the healthcare workforce in low- and middle-income countries (LMIC) [15]. While widely used in HIV, maternal, child and mental health, there is little literature to support their use in the context of NCDs in Africa, where decentralisation of NCD care to primary health centres is needed.

Our study aimed to: 1) establish a ToT cascade intervention using tailored evidence-based clinical guidelines for the diagnosis and management of hypertension, diabetes, and cardiovascular risk; and 2) build a cohort of trainers able to scale up the training model countrywide.

### Strategy and Global Health Partnership

Our strategy encompassed the process of designing and implementing a ToT programme in a LMIC, using a global health partnership that involved Mozambique’s Instituto Nacional de Saúde (INS) and Primary Care International (PCI) [16], based in the United Kingdom. INS is the national health surveillance and research implementation institution that holds the Mozambican Ministry of Health’s (MoH) mandate to address the country’s strategic priorities for the health sector and support policy design. Through its **Hy**pertension and Associated **Risk** Factors Management (HyRISK) program, INS goals are to train non-specialists to support decentralization of NCD care, promote early detection and risk stratification of HTN and DM, integrate hospital care within an appropriate referral system, and foster linkage to the community. PCI is a social enterprise specialising in healthcare capacity building to improve the quality of care of NCDs at the primary care level in LMIC and supports context-specific adaptation of pragmatic evidence-based guidelines and local cascade training, based on its experience of working with the World Health Organization (WHO) and the United Nations High Commissioner for Refugees (UNHCR), as well as with governments and Non-Government Organizations (NGOs).

**Figure 1:**
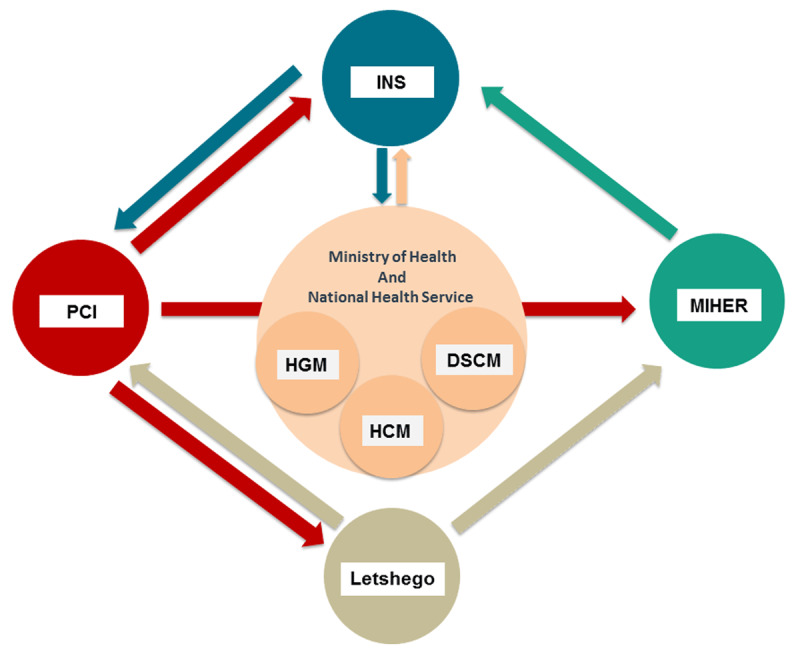
**Global Health Multilateral Partnership showing stakeholders involved in the project from Mozambique and the UK.** Letshego provided the local sponsorship for the project via a long-term partnership with Primary Care International (PCI). The Mozambique Institute of Health Education and Research (MIHER) acted as the administrative recipient of the PCI/Letshego funding. INS is the research and implementing partner and will have a role in promoting incorporation of research results into policy. The Ministry of Health – through the National Health Service – is the owner of the health facilities and will be the future adopters. Abbreviations: DSCM – Maputo city health directorate, HCM – Maputo Central Hospital and HGM – Mavalane General Hospital.

The INS NCD Division and PCI partnership was built to implement the training component of the HyRISK programme (Figure 1), supported by an ongoing PCI Health Innovation Programme sponsored by Letshego Group Limited, a financial institution headquartered in Botswana and with branches across Africa, including in Mozambique. The Mozambique Institute for Health Education and Research (MIHER) was invited to manage the program, particularly the funding.

### Setting

The study was implemented in a poor sub-urban community of Mozambique’s capital, Maputo, where INS has its clinical research site – located at Mavalane General Hospital (MGH), a public hospital with eight satellite health centres (SHC), which serve a population of approximately 800,000 people.

## Staged Approach

**Figure 2:**
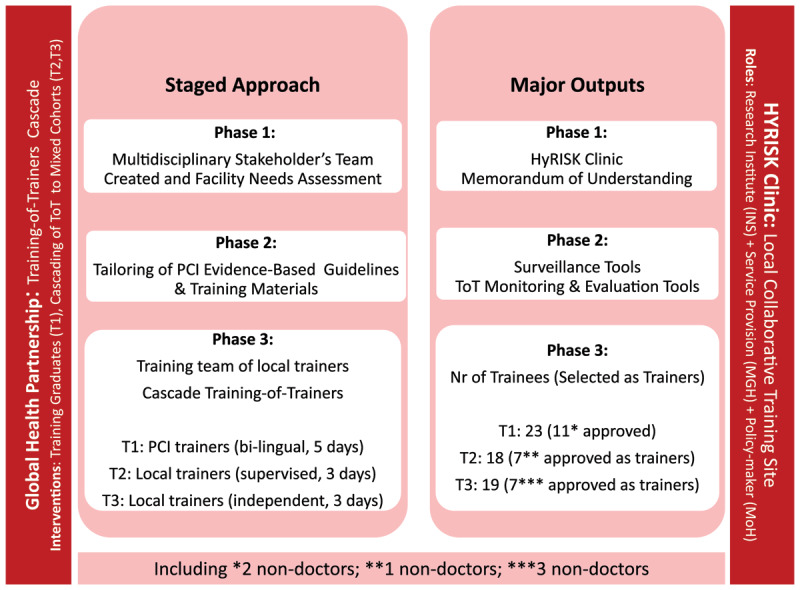
**Staged approach and major outputs of Global Health Partnership.** The strategy incorporated a global health partnership that contributed to knowledge transfer in training of evidence-based guidelines and to the creation of a collaborative training site. Contributions from the technical partner in the different stages of the project and main outputs are shown. Abbreviations: INS – Instituto Nacional de Saúde; MGH – Mavalane General Hospital; MoH – Ministry of Health; PCI – Primary Care International; T1 – Trainees Cohort 1; T2 – Trainees Cohort 2; T3 – Trainees Cohort 3.

The activities were undertaken between 1^st^ of October 2018 and the 31^st^ of March 2020. A staged approach was adopted to implement the project as outlined in Figure 2.

Phase 1, Involved establishing an in-country multidisciplinary stakeholder team, the backbone consisting of permanent general practitioners from INS/MGH, supported by trained non-physicians involved in NCD care, a pharmacist and public health workers. Three local specialists (cardiologist, internist and epidemiologist) were invited as advisors for a priority setting exercise. HTN – the most important risk factor for heart failure [17] and stroke [18] – and commonly associated DM [19] were considered the major priorities for our training model, with their complications included so that frontline workers are able to identify and refer patients in need of higher levels of care. INS’ assessment of the preparedness of MGH for HTN and DM care provision, previously done using an adaptation of the WHO Service Availability and Readiness Assessment survey [20], had revealed the lack of clinical guidelines for management of HTN [12] and DM as a critical gap.

**Figure 3:**
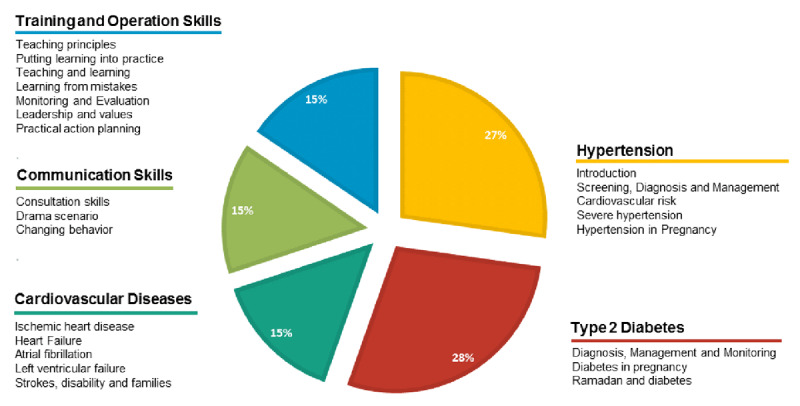
**The content of the training curriculum in the workshops.** Shown with the pie area corresponding to the proportion of time allocated to each theme. The training package duration was 24 hours spread over 8 hours daily. This includes time spent on participant registration, adequate refreshment breaks, monitoring and evaluation quiz, post course evaluation questionnaire, certificate presentation and taking of group photograph. See Appendix 1 for more detail.

In Phase 2, PCI training materials and evidence-based clinical guidelines were jointly tailored to the Mozambican context, based on the WHO PEN [21] and the Mozambique essential medicines list [22]. A workshop to harmonize these adaptations was held with the presence of (1) MoH officials representing the National Directorates for Hospital Services, Public Health, Essential Medicines, and Health Education; (2) Maputo City health authorities including MGH leadership and representatives of its referral site (Maputo Central Hospital); (3) Mozambique WHO office representatives; (4) selected expert clinicians – cardiologists, pulmonologists, internists, and gynaecologists; and (5) general practitioners and non-physicians who act as frontline health workers for diagnosis and management of HTN and DM, namely medical officers, trained nurses, pharmacists and preventive medicine health professionals. The training curriculum included screening, risk assessment, diagnosis, and management of HTN, DM and cardiovascular disease; with additional topics on communication, operational and training skills (Figure 3). Local leadership ensured institutional support for the program, and enabled participants to attend during working hours. In addition, two of the three local clinical experts (internist and cardiologist) provided continuous technical support at no cost to the project.

The following stage (Phase 3) consisted of building the initial team of local trainers (Training 1 [T1]). Two senior PCI trainers delivered the first training workshop (32 hours spread over four days) to 23 frontline doctors working in primary health facilities and MGH, who foresaw being in the same role for at least one additional year. The workshop was delivered in English with two trainees translating the content, comments and questions to/from Portuguese to/from English; participants were encouraged to contribute and discuss in both languages. After each session participants reached consensus on the content and style of the training materials. Any further adaptations resulting from the discussion were used to guide future development of training materials and support programme implementation. All 23 participants successfully completed the programme.

Following the training, three meetings were organized with technical support from the two local specialists, to assess the expectations of the future local trainers and decide on the ways forward in a participatory manner. They were asked about their willingness to become local trainers, commitment to participate in weekly sessions to enhance their skills, and desire to allocate time to prepare and deliver the training materials to their peers, in preparation for the starting of the cascade training. Fourteen trainees agreed; six declined due to work overload and three had personal reasons for not accepting the commitment (move to the private sector, maternity leave, and new professional commitments). Communication between group members was established through a WhatsApp group, to exchange details of scheduled meetings, training program and member’s roles; reminders were also sent three days before and on the day of the meeting to promote participation.

Over the following 42 weeks, the INS team and the consultant specialists led 20 bi-monthly meetings for the 14 trainers-to-be. Each participant had the opportunity to prepare and deliver two sessions, which used role-play, motivational ‘games’ and exercises. To encourage networking, people were seated informally, and background music was provided to promote interaction during breaks when a light meal was served. Eleven trainers showed commitment, dedication, and mastery of the training content, and were therefore approved to become local trainers of trainers. In preparation for the first cascade training, they benefited from group and individual distance-mentoring sessions with the senior PCI trainers in the UK, held via Skype. The clinical guidelines and training materials were translated into Portuguese using technical support from PCI, and the new knowledge and clinical skills were put into practice through supervised clinical work at the HyRISK clinic. The three participants who were unable to attend regularly left the group.

### Implementation of the Training of Trainers Cascade

**Figure 4:**
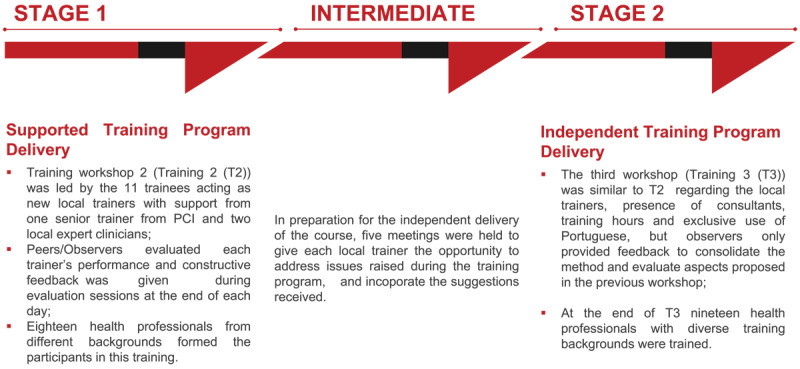
**Implementation of Training of Trainers cascade.**

The cascade of training included two additional workshops led by the local team, with supervision and evaluation from the expert clinicians and trainers. As the sessions were planned for delivery in Portuguese, and to reduce the time that healthcare workers needed to be released from their clinical work, the course was adapted and reduced to 24 hours spread over three days (Figure 4).

### Major Outputs

The major outputs of the project are outlined in Figure 2 and included 1) the development of a HyRISK clinic run by INS trainees within MGH to receive referrals from all other trainees working in the study area; a memorandum of understanding between all stakeholders to share results and ways forward, 2) surveillance tools which were developed for program monitoring and evaluation by INS and 3) a cadre of local master trainers.

Between February 2019 and February 2020, the HyRISK clinic provided an environment for consolidation of classroom training and was used to pilot the new local clinical guidelines. The clinic was staffed five days a week, eight hours per day by two doctors, one medical officer, two nurses, and a service assistant. Following referral to the clinic from health facilities in the study area, 15–30 minutes appointments were scheduled by the nurse using mobile telephone, with same day laboratory blood tests to reduce the cost of unnecessary visits to the hospital. Adapted HyRISK/PCI clinical guidelines were used to aid patient care.

### Development of Evaluation Tools for Training workshops

Three different methods were used to reflect the knowledge acquired, self-reported enhanced clinical skills, and confidence gained by the local trainers and their trainees (Table 1).

**Table 1:** Evaluation Tools for Training Workshops.


TOOL	DESCRIPTION

Knowledge & Self-Reported Clinical Skills and Confidence.	Knowledge gained during training workshops was measured using anonymized pre- and post-training assessment quizzes completed by participants. The quiz included 10 multiple-choice questions relating to clinical knowledge of HTN, DM and their cardiovascular complications. Pre- and post-course clinical skill confidence in examination of the diabetic foot, and counselling on smoking cessation was measured using a 5-point Likert scale (1 – no confidence, 5 – very confident).

Participant’s End of Course Evaluation of Training Activities Delivered by Local Trainers.	Detailed end of course evaluation questionnaire was completed confidentially by participants in training workshops T2 and T3, to understand how the training was viewed by the first-time participants trained by the new local trainers.

Program Evaluation by Local Trainers.	At the end of T3 an anonymous feedback survey was obtained from the new local trainers using written questionnaires; the aim was to assess how these new trainers evaluated their experience, assessed the training process, and self-reported their skills and confidence in training, again using a 5-point Likert scale.


Additionally a conceptual framework for training of trainers interventions in global health designed by Mormina et al, was used to structure the lessons learnt from the programme [15]. The framework designated ‘**TRAIN**’ considers five key elements for achieving a successful and sustainable ToT model: ‘**T**alent’ of trainees/trainers, ‘**R**esources’ necessary, ‘**A**lignment’ with health policy, ‘**I**mplementation’ or planning, and ‘**N**urturing’ of the training cascade.

#### Ethical Issues

The Institutional Review Board at the Faculty of Medicine, Eduardo Mondlane University and Maputo Central Hospital, Mozambique (reference number IRB 00002657) granted ethical approval for the study implementation. Informed consent was obtained from participants in the programme.

## Results

During 18 months we trained 60 health professionals (51 female), from expert clinicians to non-physicians of different backgrounds, and 11 general practitioners were certified as new local trainers of trainers. The HyRISK dedicated clinic assisted 490 patients with a mean age of 60 years, of whom 88% were female. Severe and/or complicated HTN was found in 119 (24%) patients, of whom 13 (11%) had DM as a comorbidity; additional relevant comorbidities included cerebrovascular accident (25 patients; 21%), renal insufficiency (9; 7.6%), heart failure (5; 4.2%) and atrial fibrillation (2; 1.7%). Furthermore, infectious diseases were common, with 22/119 patients living with HIV (18.5%) and 4/119 having active tuberculosis (3.4%). Five patients died between February 2019 and February 2020 of all-cause mortality.

*Impact of COVID-19*: Following the advent of COVID-19, restrictions to patients’ mobility and discontinuity in chronic disease care was noted (excluding for HIV/TB). A further 16 patients died between April 2020 and May 2021, all with at least one other chronic disease, namely HIV (8/16), cerebrovascular accident (5/16), diabetes (5/16), renal insufficiency (2/16) and tuberculosis (1/16).

### Knowledge & Self-Reported Clinical Skills and Confidence

Overall pre- and post-test scores improved in all training workshops, from 53% to 90%, 59% to 78%, and 58% to 74% in training workshops T1, T2 and T3 respectively (Table 2). The participants reported a mean improvement in confidence (Likert scale 1-5) to perform diabetic foot examination (DFE) from 3.0 to 4.4, and in consultation skills for smoking cessation advice from 3.2 to 4.2.

**Table 2:**
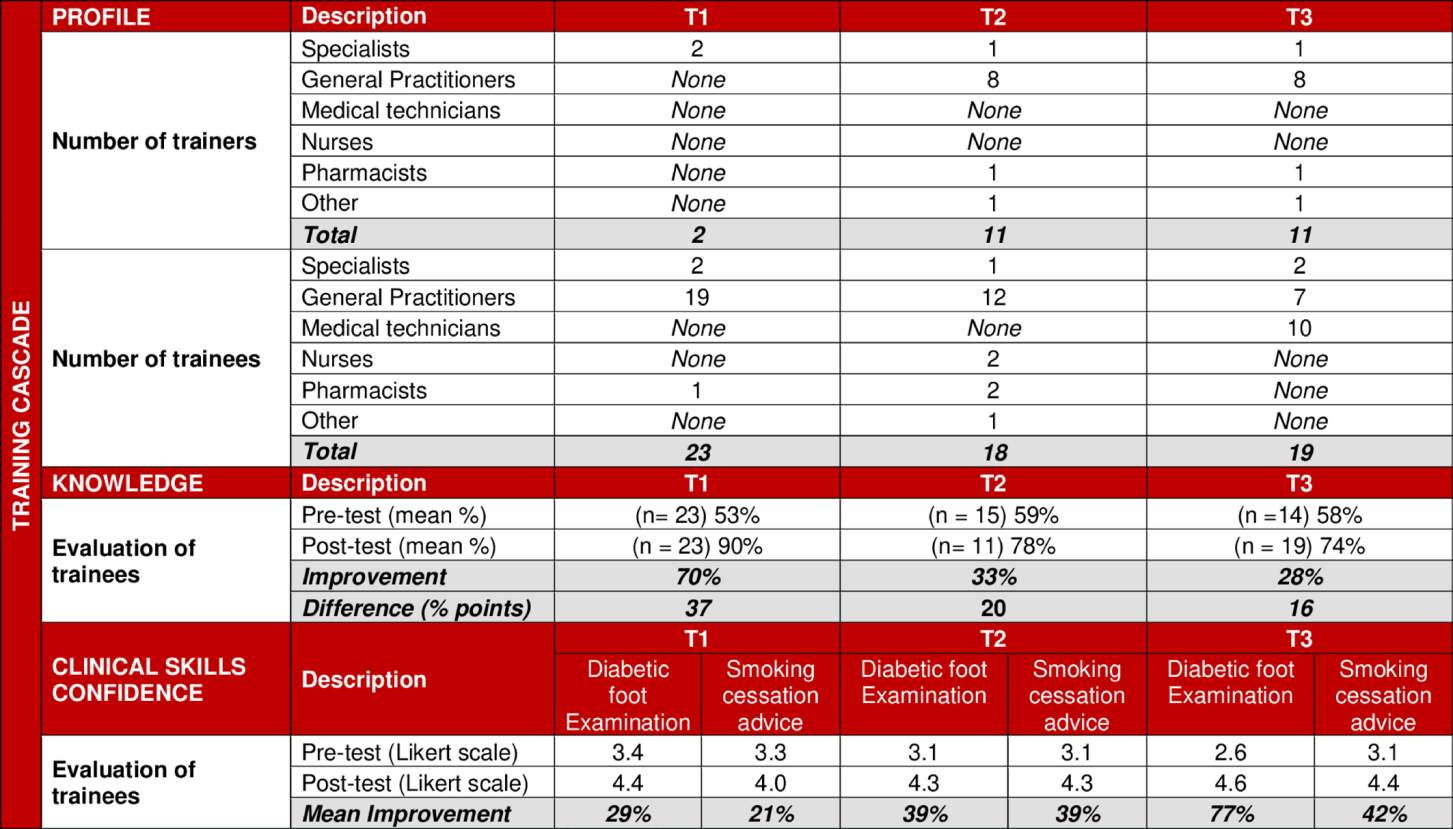
**Knowledge & Self-Reported Clinical Skills Confidence Pre- and Post-Training.** T1 – Trainees Cohort 1; T2 – Trainees Cohort 2; T3 – Trainees Cohort 3.

*End-of-Course Evaluation of Training Activities Delivered by Local Trainers*: The participants in Workshop T2 reported that the training was ‘extremely useful’ (n = 18): 90% for DM and 80% for HTN. Regarding clinical skills training for DFE and counselling for smoking cessation, 50% and 70% respectively reported the training as being ‘extremely useful’. When asked how much they would use the skills obtained, 62.5 % of participants said that they will now do foot assessment in all diabetes patients, and 28.6% said they were planning to be more empathetic with patients in consultations. On average participants rated the course at 9.7 points out of 10.

Workshop T3 participants considered the training ‘extremely useful’ (n = 19): 87 % for DM, 82% for HTN and 66% for clinical skills in DFE. While 87% of the participants said they would do DFE in all patients who have diabetes in the future, 61.1% said they would be more empathic to patients in consultations, namely greet all patients, trying to be more attentive to the patient’s complaints (mentioned or shown), listening to the patients more and treating them with care (affection, respect, and consideration). On average participants rated the course as 9.6 out of 10.

*Program Evaluation by Local Trainers*: Out of the 11 local trainers, one did not complete the evaluation form. Ten local trainers reported confidentially, that they greatly enjoyed the training programme (mean Likert scale 4.9/5) and felt they had achieved its learning objectives (mean Likert scale 4.6/5). All respondents felt they were more confident as trainers with confidence levels in training improving from a mean Likert scale of 3/5 pre-training to 4.8/5 post-training. Nine reported improvements in knowledge and approach to patients with the targeted conditions, and six felt they had also improved their communication skills. Finally, all trainers recognised that the training would improve patient care and the need to extend the program and replicate the training to other areas of the country, while nine said they would recommend the training program to other colleagues. Six trainers had thought about giving up the course – five due to work overload; two suggested the introduction of a financial incentive for trainers, while one wanted some sort of formal professional recognition. Table 3 describes the challenges and lessons learnt during the implementation of the program.

**Table 3:** Challenges and Lessons learned, presented alongside the five key elements of the TRAIN* framework to promote ToT sustainability (*TRAIN = T – Talent, R- Resources, A-Alignment, I-Implementation, N-Nurture) [15].


	CHALLENGES	DESCRIPTION	LESSONS LEARNED

T	Engagement of TRAINERS	Due to conflicting clinical priorities and heavy workload, there is high risk of low motivation and poor retention in training.	Create supportive and friendly training environment with time for networking, music and provision of food; Organize regular reminders and communication via WhatsApp, google calendar, SMS or mobile phones; Consider small financial incentives for those coming from far, and include this cost in the budget; Involve major stakeholders to guarantee training as a priority and avoid absences due to clinical duties; Provide professional development support and mentorship by including senior clinicians through the program; Use this activity as part of continuing professional education and include in certification; Incoporate soft skills and capabilities of trainers into training to adopt an ethos of improved quality of care.

	Diverse background of TRAINEES	Due to extreme shortage of trained doctors in Mozambique, all types of non-physicians and mid-level clinicians who provide frontline care in primary health facilities, were involved in the training cascade.	Adopt criteria for trainee selection, valuing enthusiasm, personal commitment and career expectations; Select trainees according to their leadership and communication skills, in addition to technical competence; Involve trainees in scheduling of meetings to avoid major schedule constraints; Include different cadres to allow broader understanding of health system challenges and sense of common goal and encourage collaboration between different cadres to achieve effective task sharing within health facilities; Consider building smaller groups according to knowledge levels and skills, to encourage peer-to-peer support; Consider developing new modules for specific areas where the goals may differ for specific health workers.

R	Logistical and administrative challenges	Resources and administrative procedures required for training include change in timetables and displacement from their health facilities to the training site.	Choose a central location for training of a given catchment area and offer travel vouchers if necessary; Ensure the budget includes provision of food/refreshments to allow full participation after clinical work; Include a checklist of computer, projector, printing capacity and stationery to ensure a good learning environment; Obtain authorization from high level administrative leadership in advance to free trainees’ time schedule; Whenever possible, provide administrative support to the training team.

A	Alignment with local health policy	There was a need to align the training content with local health policies, task-shifting strategies, and structure of the national health system.	Cascade plans and certification need to be discussed with health authorities to be part of individual development; Ensure that the different health care workers within the system are involved to ensure integration of care; Involve non-clinicians (laboratory, pharmacy, procurement) in the initial modules to strengthen team work; Adapt clinical guidelines with prescribing authority regulations for different type and profile of health workers; Acknowledge and incorporate in the guidelines the diversity of primary health care teams within a given setting; Ensure that trainers and trainees are aware of the local strategic plans and set priorities in an inclusive manner.

I	Implementation in a different context and environment	For implementation, adaptations to the content of guidelines and training materials were needed, due to different drug availability, level of frontline health professionals and prescription norms.	Guarantee local ownership of the program for implementation of cascade training; Recognize and address unique challenges through open discussion with local stakeholders; Consider the implementation of a pilot clinic alongside the training program to highlight operational issues early; Engage the participants in the process of incorporating new clinical guidelines into the clinical setting; Use clinical outcome data and qualitative analysis to strengthen evidence to inform change.

N	Nurturing the program to ensure sustainability	In the context of high service demand and under-resourcing of the health services there is a risk of suspension of the program once the external support ends.	Ensure 360 degree evaluation of the cascade training to increase skills and confidence of trainers and trainees; Provide continuous coaching of new local trainers and provide learning resources to maintain programme quality; Leverage local partnerships to obtain buy-in and additional financial and logistical support for the program; Liaise with local health authorities and academic institutions to ensure program adoption after external funding; Ensure data on training skills gained and lessons from cascade training implementation are collected; Data collection systems should be planned for future communication with local health information systems.


## Discussion

Here we describe the design, implementation, and outputs of a ToT cascade intervention in a LMIC with a severe workforce shortage. We focused our initial efforts on HTN, DM and CV risk with excellent results in improved knowledge and self-reported clinical skills of the participants, which persisted in the cascade of training. Moreover, the participants trained by the local trainers rated the course at 9.7 and 9.6 points out of 10 for the supervised and independently delivered training workshops respectively, supporting the feasibility and acceptability of the ToT cascade model. New local trainers reported enjoying the training programme greatly (mean Likert scale 4.9/5), achieving the learning objectives (mean Likert scale 4.6/5) and feeling more confident as trainers (mean Likert scale 3/5 pre-training to 4.8/5 post-training). Although we recognise the limitations of self-reporting bias of confidence ratings, this is likely to represent a positive experience of the trainers and on-going engagement in the cascade programme.

In SSA, undiagnosed and untreated HTN is one of the largest drivers of NCD, and thus a roadmap for its prevention and control has been designed [23]. SSA is not on target in its response to the call by the WHO to reduce premature deaths from NCD by 25% by the year 2025 (25 × 25) [24]. Unique elements of this work such as using a global health technical partnership supported by a funder based in the LMIC, promoting community engagement in defining the priorities for training, targeting healthcare workers beyond medical doctors, establishing local ownership and leadership, and incorporating the training into the clinic setting, should be used to contribute to operationalize the roadmap for HTN prevention and control in Africa.

The Pan-African Society of Cardiology (PASCAR), in partnership with several organizations, including the World Heart Federation, have developed an urgent 10-point action plan to improve detection, treatment and control of HTN in Africa, one of which is ‘to promote a task-shifting/task-sharing approach in the management of HTN’ [23]. An evidence-based learning, up-to-date curriculum and on-the-job training course tailored to the African context has been developed and is to be offered on a modular basis [25]. In preparation for the introduction of NCD clinics in Mozambique, we used PCI’s long experience of training primary care health workers, as an opportunity to focus our initial efforts to train our diverse workforce on diagnosis and management of HTN and associated risk factors.

Our results showed marked improvement in knowledge for doctors during the initial training, however for the mixed cohorts taught by local trainers, composed of doctors, mid-level healthcare workers and nurses, the results were less impressive. This could be explained by the diversity of professionals receiving the training and to the lack of experience of the trainers. Despite the lower percentage in improvement of knowledge gained obtained by the mixed cohorts, a significant improvement in confidence to perform clinical skills of diabetes foot examination and smoking cessation advice was seen. Overall, these results are lower than those found a study in Ghana [26] however the conditions of the training of trainer cascades are difficult to compare.

There is scarcity of information regarding the results and sustainability of task shifting of hypertension care to non-doctors. A systematic review looking at task shifting for non-communicable disease management in low and middle income countries concluded that it is a viable and successful model, that is potentially cost effective and clinically effective [27]. However, in South Africa, a programme designed to support and expand nurses’ role in NCD care, comprising educational outreach to nurses and a clinical management tool with enhanced prescribing provisions, showed that treatment intensification rates in intervention clinics were not superior to those in the control clinics in HTN and DM during 14 months of follow-up [28]. It was highlighted however that clinical outcome improvements can be difficult to measure in the real-life clinical setting and additional research is needed to assess how this intervention translates into improved clinical outcomes. Incorporating training into clinical practice however, and training healthcare workers at their site of work, as was done via the HyRISK clinic in our programme with expert oversight, has been shown to increase the effectiveness of training strategies to improve healthcare provider practices in LMIC [29]. Furthermore, the HyRISK clinic activity was interrupted at the onset of COVID-19, with significant impact on access and delivery of chronic disease care during the pandemic [30,31], including several deaths between April 2020 and May 2021.

Like other global health collaborations we were able to create tailored support for national NCD policy and programme implementation [32], promote global standards to develop primary health care guidelines, and create a ToT cascade to support the successful adoption of evidence-based guidelines for HTN, DM diagnosis and CV risk management. Innovations to nurture the ToT intervention towards sustainability included the design of locally agreed clinical guidelines and training materials relevant to the context, an intensive support and strong local ownership, the engagement of leadership, and continuous empowering of healthcare workers over time. Qualitative research exploring the perspective of health workers and service users is necessary to help understand the barriers and facilitators for long-term impact of the training cascade on quality of care. Provision of essential medicines and clinical equipment, changes to prescribing authority and improved operational aspects of health care delivery for NCDs are also necessary for sustainability [27,33], and must be added to our innovative approach to allow successful adoption within the local health system.

## Conclusion

Our context specific interprofessional training model was successful in cascading knowledge and skills acquisition, and in establishing the initial cohort of trainers of trainers to scale up HTN, DM and CV risk management to non-doctor frontline health workers countrywide. Intensive local supervision and hands-on training empowered a new cohort of trainers to strengthen the prevention and control of cardiovascular disease. While not generalizable to all countries in the region, our approach can potentially be adapted to contribute to the concerted efforts of academics, researchers and policymakers in SSA, to address the progress along the regional roadmap for arterial hypertension prevention and control, improve the quality of care of patients with HTN and DM and ultimately reduce the burden of cardiovascular disease.

## Additional File

The additional file for this article can be found as follows:

10.5334/gh.1052.s1 Appendix 1. Adapted curriculum learning objectives for the HyRISK Training of Trainers course.
