# Supplementary material for: Empowering Frontline Primary Healthcare Workers in a Global Health Partnership Training of Trainers Intervention to Strengthen the Prevention and Control of Cardiovascular Disease in Mozambique
Source: Glob Heart. 2022 Aug 2;17(1):51. doi: 10.5334/gh.1052 (PMC9354556; doi:10.5334/gh.1052)
Supplement: Appendix 1. — Adapted curriculum learning objectives for the HyRISK Training of Trainers course. [file gh-17-1-1052-s1.pdf]

# ADAPTED CURRICULUM LEARNING OBJECTIVES FOR THE HYRISK TRAINING OF TRAINERS COURSE IN THE DIAGNOSIS, ASSESSMENT AND MANAGEMENT OF HYPERTENSION, DIABETES AND CARDIOVASCULAR DISEASE IN PRIMARY CARE

The PCI training package was adapted to the local setting with the local multidisciplinary stakeholder team and included 24 sessions, using a combination of pedagogical approaches: interactive lectures delivered on power point slides**,** focus group discussions, case/problem-based learning and discussion, and role plays for practical training and consultation skills.

## 1. INTRODUCTION

- Describe the epidemiology of non-communicable diseases globally, in sub-Saharan Africa and in Mozambique, and understand the burden of disease on the population and health care system
- Discuss the different approach needed to manage acute and chronic disease, communicable and non-communicable diseases from the clinician and patient perspectives.

## 2. HYPERTENSION

### a. Screening, Diagnosis, Assessment and Management

- Understand who should be screened for hypertension
- Understand the definition of hypertension and how to ensure accurate diagnosis
- Describe and discuss the primary and secondary causes of hypertension
- Describe the appropriate examination and investigations that should be undertaken in a newly diagnosed hypertensive patient
- Understand the main drug treatments, along with their advantages and side effects, used in the management of hypertension based on the WHO and Mozambique essential medicines list, according to PCI and HyRISK adapted clinical guidance
- Clinical case-based discussion in small groups and plenary on the management of hypertension in practice.

### b. Overall Cardiovascular Risk

- Understand the definition of cardiovascular risk
- Learn about the importance of cardiovascular risk assessment and how to make such and assessment
- Learn how to calculate cardiovascular risk using WHO risk charts
- Understand the need for primary and secondary prevention of cardiovascular disease according to PCI and HyRISK adapted clinical guidance

- Clinical case-based discussion in small groups and plenary on the management of cardiovascular risk in practice.

### c. Severe Hypertension

- Understand the pathophysiological consequences of acute and chronic severe hypertension, and how to recognise hypertensive urgency and hypertensive emergency
- Learn how to evaluate for clinical signs of end organ damage
- Explain the management of severe hypertension using PCI and HyRISK adapted clinical guidance
- Clinical case-based discussion in small groups and plenary on the management of severe hypertension in practice.

### d. Hypertension in Pregnancy

- Define hypertension in pregnancy
- Understand the possible causes of hypertension in pregnancy
- Understand how to assess and manage a patient with hypertension in pregnancy using PCI and HyRISK adapted clinical guidance
- Clinical case-based discussion in small groups and plenary on the management of hypertension in pregnancy in practice.

## 3. TYPE 2 DIABETES MELLITUS

### a. Diagnosis, Assessment, Management and Monitoring

- Understand how to define and diagnose diabetes and recognise the symptoms
- Understand the appropriate examination and investigations that should be undertaken in a newly diagnosed patient with diabetes
- Consider how to explain diabetes and its treatment to a newly diagnosed patient
- Identify the risk factors for the development of type 2 diabetes mellitus
- Learn about the main complications of diabetes, how they can be prevented and managed
- Learn about the main drug treatments for diabetes, along with their advantages and side effects
- Explain the treatment and management of type 2 diabetes in patients with hypertension according to PCI and HyRISK clinical guidance
- Learn about the importance of cardio-vascular risk assessment and how to make such an assessment in a patient with diabetes
- Discuss the management of diabetic emergencies
- Learn and practice the examination of the feet in patients with diabetes
- Clinical case-based discussion in small groups and plenary on the management of diabetes in practice.

## b. Diabetes in Pregnancy

- Describe the impact of gestational diabetes on the pregnant woman
- Learn how to diagnose and manage diabetes in pregnancy according to PCI and HyRISK clinical guidance
- Presentation of clinical cases for group and plenary discussion on the management of diabetes in pregnancy

## c. Ramadan and Diabetes

- Examine the problems faced by diabetic patients during the month of Ramadan
- Learn how to advise a patient with diabetes so that they can stay healthy and minimise risks of fasting during Ramadan

# 4. CARDIOVASCULAR DISEASES

All sections below also include a presentation of clinical cases for small group and plenary discussion;

## a. Ischemic heart disease

- Describe the different types of chest pain and its differential diagnoses
- Describe how to recognise ischaemic heart disease and its risk factors
- Discuss the treatment of ischemic heart disease and its secondary prevention.

## b. Heart Failure

- Recognize the signs and symptoms of heart failure and discuss its underlying causes
- Understand the classification of heart failure according to New York Heart Association (NYHA)
- Understand the diagnosis and treatment of heart failure in primary care.

## c. Atrial Fibrillation and Left ventricular failure (LVF)

- Understanding how to detect atrial fibrillation and left ventricular failure, and recognise the symptoms
- Understand how to manage a patient with atrial fibrillation and LVF in primary care.

## d. Stroke

- Explain what a cerebrovascular accident is
- Differentiate between transient ischemic attack and cerebrovascular accident
- Explain how to identify signs and symptoms of stroke and understand the pathophysiology
- Discuss immediate management of a patient during and after stroke
- Discuss secondary prevention of stroke.

## e. Disability and families

- Understand the signs of depression in a patient after stroke and how to proceed
- Discuss strategies for inclusion of socio-family support of stroke patients
- Reflect on the differential approach to stroke patients during consultations.

# 5. COMUNICATION SKILLS

## a. Consultation skills

- Be familiar with a simple theoretical model of the consultation
- Understand how good consultation skills can improve diagnostic accuracy and build a good rapport between clinician and patient
- Have observed some consultation techniques
- Have practice using the model and techniques in a role play
- Reflect on why consultations go wrong and the consequences
- Discuss the importance of empathy and body language to achieve better results.

## b. Changing Behaviour

- Review the epidemiology of smoking habits worldwide, in sub-Saharan Africa and in Mozambique
- Understand why people smoke, why they want to stop smoking and why it is difficult to stop smoking
- Know the main risks of smoking, and the health benefits to those who quit
- Understand the 5 stages of change and what kind of interventions can be made
- Discuss nicotine replacement therapy.

## c. Drama scenario

- Small group role play activity to include patient, health professional and observer who will act out the smoking cessation counselling session in its different stages of change. Presentation and discussion in plenary.

## 6. TRAINING AND OPERATIONAL SKILLS

### a. Teaching principles

- Understand some of the principles behind adult teaching and learning
- Conduct a practical exercise to create a weekly food menu for a patient with diabetes on a realistic budget
- Reflect on the challenges patients face in choosing foods for cooking at home and their preferred food preferences
- Introduce dietary guidance for weight reduction in patients with hypertension and diabetes
- Discuss the adoption of long-term healthy eating habits from the health professional and family perspective.

### b. Putting learning into practice

- Practical exercise to consider what has been learnt, and how this knowledge and new skills will be integrated into daily practice (clinical and non-clinical)
- Discuss the challenges of implementing new habits into daily activities.

### c. Teaching and learning

- Recognise the different resources needed for acquiring knowledge
- Understand the different ways that participants learn and the training methods that can be used
- Explain the steps to be implemented in a training workshop.

### d. Learning from mistakes

- Introduce the concept of significant event analysis
- Discuss examples of situations where participants have learned from mistakes
- Discuss the strategy for identifying errors, how to avoid them and how learn from them.

### e. Monitoring and Evaluation

- Comprehend the basic principles of monitoring and evaluation

- Understand the importance and role of monitoring and evaluation in the implementation of an activity
- Understand how to use the key monitoring and evaluation tools, the Logical Framework Approach and Key Performance Indicators.

### f. Leadership and Values

- Understand what good leadership looks like
- Understand the different types of leadership and their respective approaches including advantages, and disadvantages
- Reflect on the meaning of values
- Build a personal action plan to determine steps for ongoing development of leadership and values.

### g. Practical action planning

- Identify and list problems with NCD care in the work place, weaknesses and strengths in leadership, values, and monitoring and evaluation systems
- Identify possible changes to systems and practice that will improve NCD care
- Formulate a simple and realistic plan for change that can be implemented and supported on returning to the work place
- Discuss a weekly/monthly monitoring and evaluation plan of the results and strategy to improve the quality of care for NCDs.
